# An *in silico* approach to the identification of diagnostic and prognostic markers in low-grade gliomas

**DOI:** 10.7717/peerj.15096

**Published:** 2023-03-16

**Authors:** Melih Özbek, Halil Ibrahim Toy, Yavuz Oktay, Gökhan Karakülah, Aslı Suner, Athanasia Pavlopoulou

**Affiliations:** 1 Izmir Biomedicine and Genome Center, Izmir, Turkey; 2 Izmir International Biomedicine and Genome Institute, Dokuz Eylül University, Izmir, Turkey; 3 Department of Epidemiology and Cancer Control, St. Jude Children’s Research Hospital, Memphis, Tennessee, United States; 4 Faculty of Medicine, Department of Medical Biology, Dokuz Eylül University, Izmir, Turkey; 5 Faculty of Medicine, Department of Biostatistics and Medical Informatics, Izmir, Turkey

**Keywords:** Low-grade gliomas, Transcriptome analysis, Differential gene expression analysis, Weighted gene co-expression network analysis, Diagnosis, Prognosis, Biomarkers, Bioinformatics, Systems biology

## Abstract

Low-grade gliomas (LGG) are central nervous system Grade I tumors, and as they progress they are becoming one of the deadliest brain tumors. There is still great need for timely and accurate diagnosis and prognosis of LGG. Herein, we aimed to identify diagnostic and prognostic biomarkers associated with LGG, by employing diverse computational approaches. For this purpose, differential gene expression analysis on high-throughput transcriptomics data of LGG *versus* corresponding healthy brain tissue, derived from TCGA and GTEx, respectively, was performed. Weighted gene co-expression network analysis of the detected differentially expressed genes was carried out in order to identify modules of co-expressed genes significantly correlated with LGG clinical traits. The genes comprising these modules were further used to construct gene co-expression and protein-protein interaction networks. Based on the network analyses, we derived a consensus of eighteen hub genes, namely, *CD74, CD86, CDC25A, CYBB, HLA-DMA, ITGB2, KIF11, KIFC1, LAPTM5, LMNB1, MKI67, NCKAP1L, NUSAP1, SLC7A7, TBXAS1, TOP2A, TYROBP*, and *WDFY4*. All detected hub genes were up-regulated in LGG, and were also associated with unfavorable prognosis in LGG patients. The findings of this study could be applicable in the clinical setting for diagnosing and monitoring LGG.

## Introduction

Tumors located in the central nervous system (CNS) are defined based on their cells of origin and histopathological characteristics. Gliomas are neuroepithelial and highly heterogeneous tumors originating from neuroglial progenitor cells (Forst et al., 2014; Wirsching & Weller, 2016). Gliomas represent 27% out of all primary brain tumors, and account for 80% of the malignant primary brain cancers (Wirsching & Weller, 2016).

Traditionally, gliomas are classified, according to the World Health Organization (WHO), into low grade gliomas (LGGs) (grade I and II tumors) and higher-grade gliomas (HGGs) (grade III and IV tumors) (Wirsching & Weller, 2016). The WHO also added molecular and genetic parameters that improved the diagnostics and prognostics of this type of cancer. Major changes occurred in the subcategorization of gliomas because genetic mutations and prognostic factors may vary highly (Delgado-Lopez et al., 2017; Ferris et al., 2017). Currently, aside from the traditional system, classification is done based on the type of glioma cells such as astrocytoma or oligodendroglioma, integrated with the mutations status of genes such as the isocitrate dehydrogenase gene *(IDH) 1* and *IDH2*, *ATRX* and *TP53*, as well as 1p/19q deletion (Dono et al., 2021).

LGGs generally occur in people between 20 and 40 years old. While the most common symptoms among patients is the presence of seizures, patients may have headaches as well. Primary tumor location greatly affects both the symptoms and severity of LGG. Seizures are thought to be due to the invasion of cancer tissue into the cortex region of the brain (Schiff, 2017). Although LGG constitutes 20% of all primary brain tumors, the survival period of LGG patients ranges between 4.7 and 9.8 years. Therefore, LGG patients are expected to survive longer compared to HGG patients (Kumthekar, Raizer & Singh, 2015). The most malignant form of gliomas is glioblastoma (GBM) and is recognized to be one of the deadliest brain tumors affecting adults. GBMs are classified, according to WHO, as grade III and grade IV tumors (Chen, Cohen & Colman, 2016; Claus et al., 2015).

There are different options of treatment for patients with LGG. Notably, some LGG patients are not even aware of this condition. Therefore, timely and accurate diagnosis is critically important before tumor progresses to a higher grade. Compared to the short survival rate of patients with HGGs or GBMs, patients with LGGs show longer rates of survival, which raises controversies regarding the decision of treatment administration, medication dosage and associated side effects (Cancer Stat Facts: Leukemia—Acute Myeloid Leukemia (AML), https://seer.cancer.gov/statfacts/html/amyl.html). The conventional treatment options include surgical resection, radiotherapy and chemotherapy (Rossi et al., 2019). Therefore, accurate diagnosis and prognosis are of critical importance, since inappropriate selection of treatment can result to a decrease in LGG patients’ quality of life (Carabenciov & Buckner, 2019; Tom et al., 2019).

The great advancements in high-throughput technologies allowed the generation of a great amount of LGG-relevant gene expression data (‘big data’) deposited to publicly available databases. This allowed us, in the current study, by employing an integrative and robust *in silico* methodology, diverse state-of-the-art software and stringent criteria to process, analyze and interpret publicly available LGG-relevant transcriptomic data. To this end, large-scale RNA sequencing data were exploited in order to identify genes differentially expressed between LGG samples and corresponding normal brain specimens. Differentially expressed genes detected by three different methodologies were subjected to weighted co-expression network analysis to identify genes with similar expression patterns that comprise functionally distinct modules. Genes within these modules were further correlated with important LGG clinical traits and hub genes were screened by applying network-based methods. In this way, a set of eighteen genes was revealed, which could be considered as potential diagnostic biomarkers for discriminating LGG patients from healthy individuals. Furthermore, the predictive value of these genes for the overall survival of LGG patients was investigated. Previous efforts have focused on the expression of long non-coding RNAs (Reon et al., 2016) or prognostic biomarkers (Liu et al., 2021b) in LGGs. To the authors’ knowledge, the present study is the first comprehensive and updated study, where a massive amount of data derived from three major resources was utilized towards the identification of candidate diagnostic and prognostic markers in LGG.

## Materials and Methods

**Figure 1:**
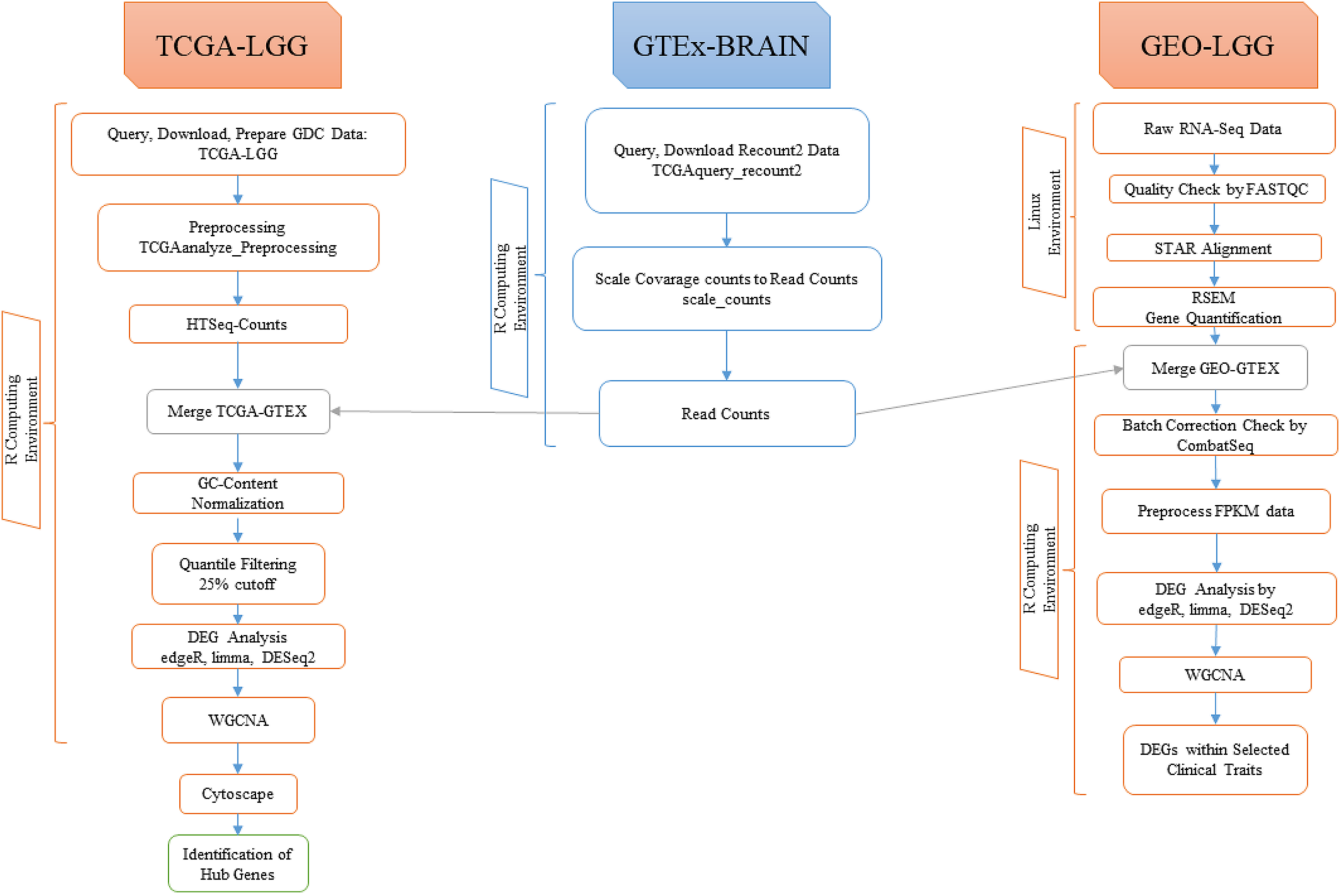
Illustration of the overall pipeline followed in this study.

A graphical illustration of the overall procedure followed in this study is depicted in Fig. 1.

All analyses were performed in the R statistical computing environment v.4.2.0 (R Core Team, 2022), unless otherwise stated.

### Data acquisition and preprocessing

#### Acquisition of low-grade glioma data from TCGA

LGG data were downloaded from The Cancer Genome Atlas (TCGA) (https://www.cancer.gov/tcga) by using the ‘TCGAbiolinks’ R package (Colaprico et al., 2016). Harmonized RNA-Seq data were downloaded with the *GDCprepare* function. A total of 529 samples were downloaded and processed *via* the GDC portal (https://portal.gdc.cancer.gov/) from the TCGA database. Then the downloaded transcriptomic data were preprocessed by using the *TCGAanalyze_Preprocessing* function of the ‘TCGAbiolinks’ package, in order to detect low correlated samples, termed ‘outliers’, which resulted to a count data matrix to be used in further steps.

#### Retrieval of brain transcriptomic data from GTEx

A total of 120 corresponding normal brain samples were downloaded from the Recount2 project (Collado-Torres et al., 2017) of the Genotype-Tissue Expression (GTEx) (GTEx Consortium, 2013) (https://gtexportal.org) database, by using the *TCGAquery_recount2* function of the ‘TCGAbiolinks’ package, as ranged summarized experiment (RSE) objects. Count data were scaled, by using the *scale_counts* function of the ‘Recount’ package.

#### Acquisition and processing of LGG data from GEO

**Figure 2:**
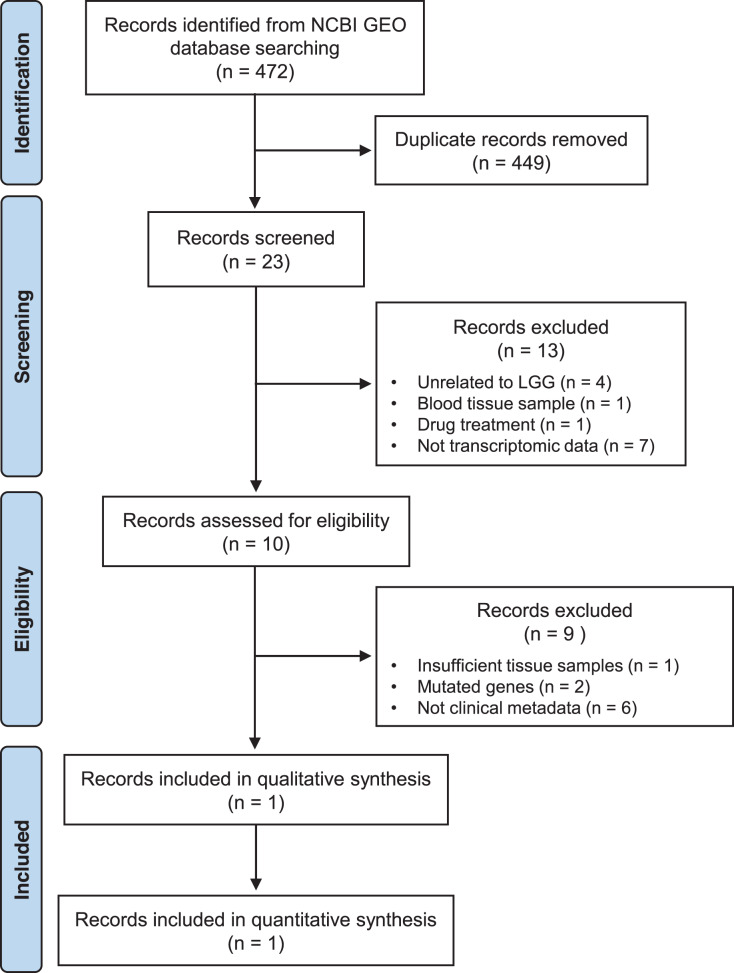
PRISMA flowchart of selecting GEO records.

The public repository of NCBI GEO (Gene Expression Omnibus) DataSets (Edgar, Domrachev & Lash, 2002) was thoroughly searched for LGG gene expression data using the keywords: (“low grade glioma” OR “low grade gliomas”) AND (“homo sapiens” OR “human”), following the PRISMA (http://www.prisma-statement.org/) guidelines (Fig. 2). In order to be considered eligible, the studies had to fulfill the following inclusion criteria: (i) human LGG tissue samples, (ii) gene expression data available, (iii) more than three LGG tissue samples, (iv) availability of clinical metadata, (v) inclusion of more than 5,000 genes in the dataset, (vi) wild-type genes. Accordingly, the studies were excluded based on the following criteria: (i) studies on animal models or cell lines, (ii) not tissue samples (*e.g*., blood), (iii) treated samples (*e.g*., drugs or radiation). Collectively, 654 relevant datasets were retrieved from GEO DataSets (up to 25 April 2022). The eligible dataset series GSE184941, which contains mRNA profiles of human low- and high-grade glioma samples, was selected for further analysis. A total of 79 LGG out of 180 samples in GSE184941 were analyzed in this study.

For this analysis, the GTEx RNA-Seq pipeline was followed (Fig. 1). FASTQ files were downloaded by using the ‘SRA Tool Kit’ v.2.11.1 (Leinonen, Sugawara & Shumway, 2011). The raw RNA-Seq reads in the FASTQ files were aligned to the GENCODE Human Reference Genome Release 39 (Frankish et al., 2021) with the aligner ‘STAR’ v.2.6 (Dobin et al., 2013). Those samples where the percentage of the uniquely mapped reads was lower than 70% were excluded, and 34 samples out of the 79 GEO-derived LGG samples were retained for subsequent analyses.

The *rsem-prepare-reference* function in ‘RSEM’ v.1.3.3 (Li & Dewey, 2011) was used for gene expression level estimation (*i.e*., quantification).

All count data of the 34 LGG samples were collected and combined into a matrix file with the corresponding sample ID and gene ID using the *rsem-generate-data-matrix* function of ‘RSEM’.

#### Merging of TCGA-GTEx and GEO-GTEx data

The preprocessed TCGA-derived LGG count data and the retrieved GTEx count data were merged based on their matching gene IDs. Merged TCGA-GTEx count data were further normalized for ‘GC content’ using the function *TCGAanalyze_Normalization* of ‘TCGABiolinks’ in the EDASeq protocol (Risso et al., 2011). Then, quantile filtering was applied with a 25% cutoff.

GEO-LGG and GTEx-extracted count data were merged based on matching gene IDs. Then, batch correction was applied by ‘ComBat-seq’ (Zhang, Parmigiani & Johnson, 2020) (https://github.com/zhangyuqing/ComBat-seq), an adjustment tool that uses negative binomial regression to detect batch effects. The results from ‘ComBat-seq’ indicated that the merged GEO-GTEx count data required no batch correction.

### Principle component analysis of transcriptomes

Principal Component Analysis (PCA) (Jolliffe & Cadima, 2016) was applied for detecting variances among glioma and normal groups. Before performing PCA, raw read counts of both merged TCGA-GTEx and GEO-GTEx data were normalized by converting them to FPKM values, with the *count2fpkm* function of ‘RNAAgeCalc’ (Ren & Kuan, 2020). The FPKM data were also filtered, so as the expression value of the individual genes was greater than ‘1’ in at least half of the samples in each group (TCGA-GTEx and GEO-GTEx). Next, the merged and filtered FPKM data were log2 transformed and the R function *prcomp* was used to generate PCA plots. For PCA plot visualization, the *fviz_pca_ind* function of the ‘factoextra*’* package (Kassambara & Mundt, 2020) was used.

### Differential gene expression analysis

To detect statistically significant differentially expressed genes (DEGs) between the ‘glioma’ and ‘normal’ samples, filtered and merged read count data with sample IDs and genes were provided as input to three software tools for conducting differential gene expression analysis (DGEA), namely, ‘edgeR’ v3.34.1 (Robinson, McCarthy & Smyth, 2010), ‘limma’ v3.48.3 (Ritchie et al., 2015) and ‘DESeq2’ v1.32.0 (Love, Huber & Anders, 2014). The output DEGs were selected based on an absolute log2 fold-change (|log2FC|) ≥ 2; the threshold for the False Discovery Rate (FDR)-adjusted *p*-value was set less than 0.05. To visualize the DEGs, heatmap plots of DEGs were generated by using the ‘pheatmap’ package v1.0.12 (Kolde, 2019).

### Weighted correlation network analysis

Weighted Gene Co-Expression Network Analysis (WGCNA) (Langfelder & Horvath, 2008) was performed of the common DEGs detected by ‘edgeR’, ‘limma’ and ‘DESeq2’ of the LGG samples derived both from TCGA and GEO. In this study, the ‘WGCNA’ R package v1.70-2 (Langfelder & Horvath, 2008) was utilized to identify highly correlated gene patterns, clusters, modules and module-LGG clinical trait relationships.

The FPKM matrix data of the detected DEGs from TCGA and GEO were log2(data+1) transformed, so as to normalize the FPKM data. The function *goodSamplesGenes* of the ‘WGCNA’ package was used to detect any possible outliers.

The *pickSoftThreshold* function of ‘WGCNA’ was used for the selection of a suitable soft-thresholding power. The adjacency matrix of gene expression data was created by using the selected power with the *adjacency* function of the ‘WGCNA’ package.

To eliminate any noise, the adjacency matrix was transformed to Topological Overlap Matrix (TOM) and then dissimilarity was calculated by using the ‘WGCNA’ function *TOMsimilarity(adjacency)* based on dissTOM = 1 – TOM. In this way, only highly correlated genes are grouped together. Next, trees of genes (*i.e*., dendograms) were created by hierarchical clustering with the *hclust* function.

Modules in the dendrograms were detected using the *cutreeDynamic* function of ‘WGCNA’. The highly correlated genes detected in the previous step were assigned into color-coded modules, and modules with similar expression profiles were detected and merged. To this end, the ‘eigenene’ (*i.e*., first principal component) of each module was calculated to estimate co-expression similarity and then were clustered again. For this purpose, the functions ‘*moduleEigengenes*’, ‘*cor*’, again ‘*hclust*’ and ‘*mergeCloseModules*’ of the ‘WGCNA’ package were used. The plot of these modules was generated by using the *plotDendroAndColors* function of ‘WGCNA’.

To visualize the weighted gene co-expression networks, heatmap plots were generated with the *TOMplot* function of ‘WGCNA’, where each row and column correspond to a gene and sample, respectively.

### Association of modules with LGG clinical traits

Clinical data of samples derived from TCGA were acquired using the *GDCprepare* function of ‘TCGABiolinks’. The clinical traits of interest from TCGA were Primary Diagnosis, Age at Diagnosis, Vital Status, Sample Type, Site of Resection Biopsy, Prior Treatment, Gender, Race, and Tissue Organ Origin.

Clinical data of samples obtained from GEO were downloaded from the GEO database (https://www.ncbi.nlm.nih.gov/geo/). Selected clinical traits for the GEO samples were Age, Progressed Tumor Pathology, IDH Mutant Status, Vital Status, Gender, Initial Tumor Pathology, and Patient Initial Grade.

The relationships between the gene modules and LGG clinical traits were investigated by using the eigengene information of each module. To this end, traits without numbers were converted to their mathematical abbreviations by using the *cor* and *corPvalueStudent* functions. Heatmaps of this analysis were generated with the *labeledHeatmap* function, and include the Spearman correlation coefficient of the modules with clinical traits and the corresponding *p*-value.

### Gene co-expression and protein-protein interaction network construction

Gene co-expression and protein-protein interaction networks were generated in this study. Cytoscape v3.9.1 (Shannon et al., 2003) (https://cytoscape.org/), an open source platform, was used for network analysis and visualization.

To this end, the adjacency matrix (*i.e*., co-expression data matrix) of genes of the selected modules were filtered by setting the threshold for the gene co-expression value at 0.5, so as to increase the robustness of the study.

Protein-Protein Interaction (PPI) network analysis was performed by providing the genes of the co-expression data matrix as input to the database Search Tool for the Retrieval of Interacting Genes (STRING) v1.7.0 (Szklarczyk et al., 2021). The direct and indirect as well as the physical and functional associations of the corresponding gene products were investigated.

The gene-gene and protein-protein association data were uploaded to the Cytoscape and the Cytohubba (Chin et al., 2014) plugin of Cytoscape, which allows network investigation by eleven different ranking algorithms: Degree method (Deg), Maximum Neighborhood Component (MNC), Density of Maximum Neighborhood Component (DMNC), Maximum Clique Centrality (MCC), Closeness (Clo), EcCentricity (EC), Radiality (Rad), BottleNeck (BN), Stress (Str), Betweenness (BC), Edge Percolated Component (EPC). In this way, the most highly connected/correlated genes/proteins in the gene co-expression and PPI networks were selected by using the node-degree filter.

### Overall survival analysis

The prognostic value of the ‘hub’ genes detected in the previous section was explored. To assess whether the expression levels of these genes are associated with the overall survival (*i.e*., a person is either alive or dead) of LGG patients, the web-based tool GEPIA (Gene Expression Profiling Interactive Analysis) (Li et al., 2021a; Tang et al., 2017) version 2 (http://gepia2.cancer-pku.cn/), as well as the Chinese Glioma Genome Atlas (CGGA) (Zhao et al., 2021) (http://www.cgga.org.cn/), were applied. GEPIA2 retrieves and analyzes survival data from the integrated TCGA pan-cancer clinical data resource (Liu et al., 2018). The LGG patient cohorts were divided into ‘high-risk’ and ‘low-risk’, by setting the cutoff values for high and low gene expression at 50%; samples with gene expression level higher/lower than the 50% cutoff are considered as high/low-expression patient cohorts, respectively. CGGA is a comprehensive repository of multi-omics data derived from Chinese glioma patients.

## Results

### Comprehensive characterization of LGG transcriptomic profiles compared to normal brain

**Figure 3:**
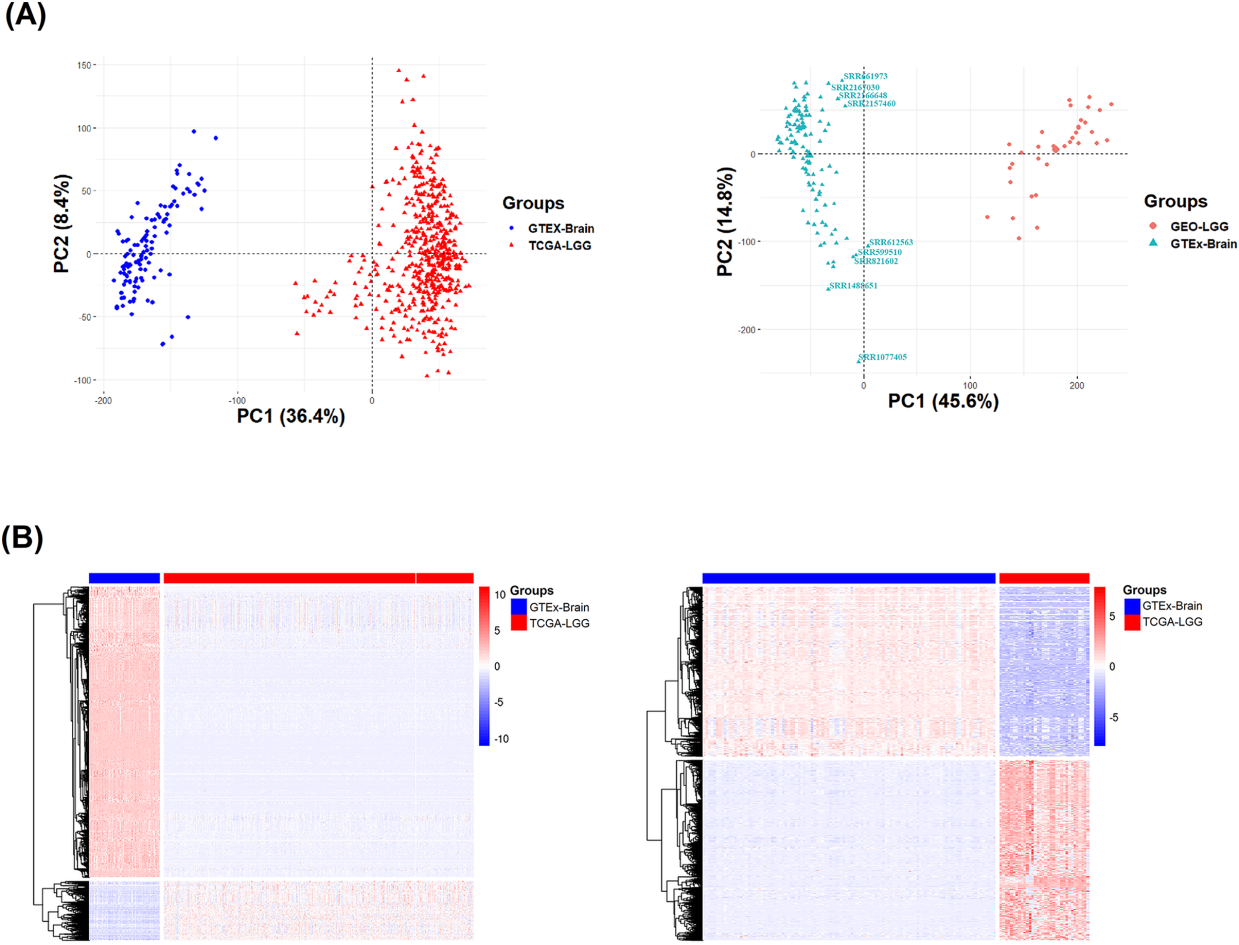
Transcriptome profiles and differentially expressed genes between TCGA and GTEx, and GEO and GTEx. (A) Principal component analysis of the transcriptomic profiles of TCGA and GTEx (left); GEO and GTEx (right). Red and blue dots represent LGG and normal samples, respectively, from TCGA and GTEx (left); GEO and GTEx (right). (B) Heatmap plots of significant DEGs generated by edgeR between TCGA and GTEx (left); GEO and GTEx (right). Each row represents a gene. LGG and normal brain samples in the top legend are denoted by red and blue color, respectively. Red and blue color in the heatmap represents up-regulated and down-regulated genes, respectively.

To eliminate any batch effects and differences, transcriptomic profiling analysis was performed on the RNA-Seq data of the LGG and normal brain samples from GTEx, as well as GEO and GTEx. To this end, the distribution of the transcriptome profiles of the 529 TCGA, 120 GTEx and 34 GEO samples were investigated *via* principle component analysis (PCA). As anticipated, the TCGA and GEO *vs* GTEx samples form separate clusters (Fig. 3A), suggestive of distinct transcriptomic profiles. In the GEO *vs* GTEx PCA plot (Fig. 3A, right), nine GTEx outlier samples were detected (SRR599510, SRR2157460, SRR612563, SRR821602, SRR1488651, SRR2167030, SRR2166648, SRR1077405 and SRR661973).

After the exclusion of outliers, DGEA was conducted using edgeR, DESeq2 and limma for detecting statistically significant DEGs. DGEA between TCGA and GTEx resulted to 804 up-regulated, 3,972 down-regulated genes from edgeR; 580 up-regulated, 4,332 down-regulated genes from DESeq2; and 709 up-regulated, 4,475 down-regulated genes from limma. Likewise, DGEA between GEO and GTEx resulted to 4,117 up-regulated, 4,382 down-regulated genes from edgeR; 4,203 up-regulated, 4,350 down-regulated genes from DESeq2; and 4,861 up-regulated, 4,090 down-regulated genes. Heatmaps of the DEGs generated by edgeR of TCGA *vs* GTEx, and GEO *vs* GTEx, are shown in Fig. 3B. Collectively, 4,465 DEGs were detected between TCGA and GTEx, including 528 up-regulated and 3,937 down-regulated genes (Fig. S1), and 7,975 DEGs between GEO and GTEx, including 3,966 up-regulated and 4,009 down-regulated (Fig. S2). In addition, there was a rather significant 60% overlap between the TCGA-GTEx and GEO-GTEx DEGs with respect to TCGA DEGs.

### Weighted gene co-expression network analysis of DEGs

**Figure 4:**
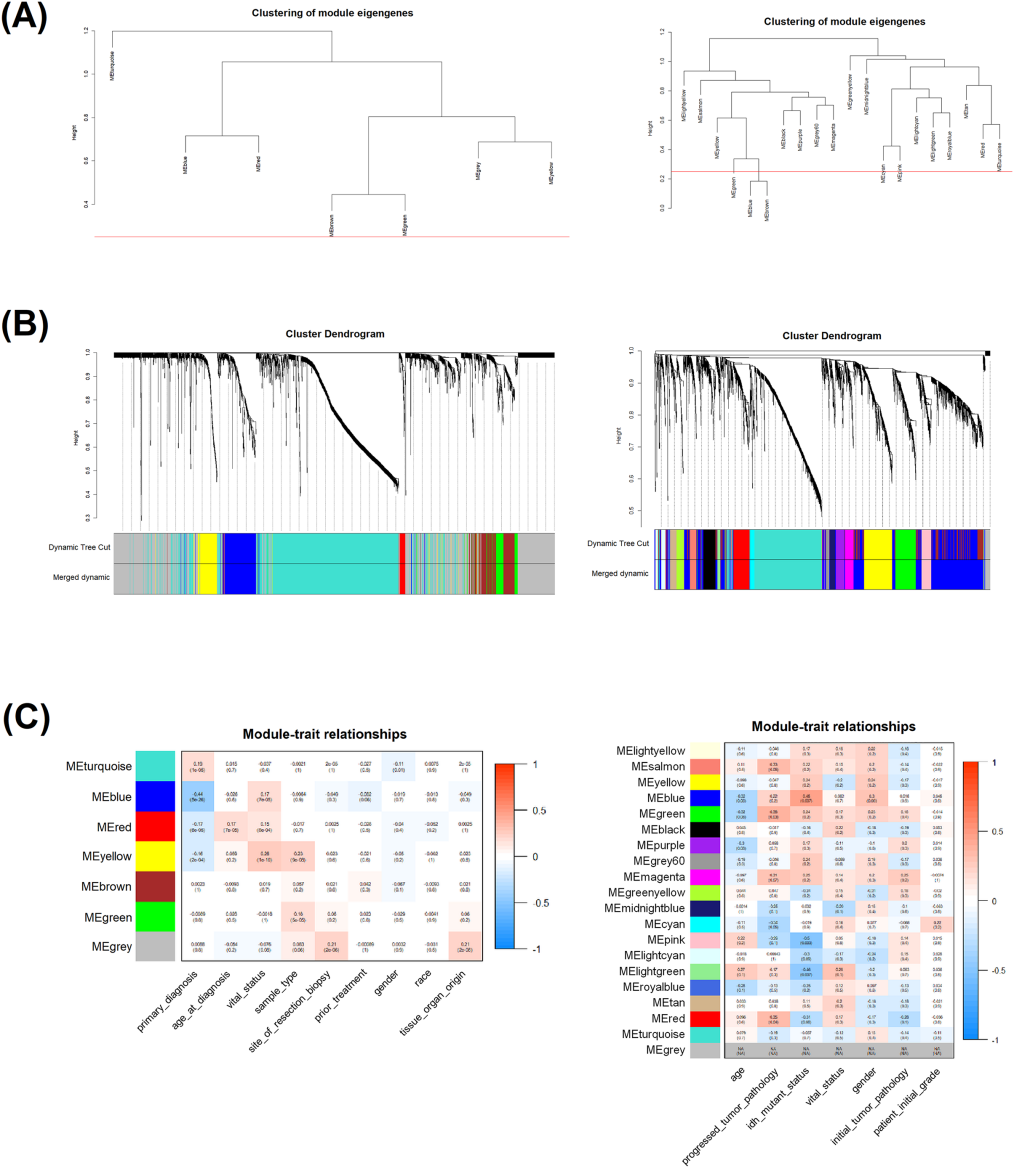
Module detection and module-clinical trait relationships by WGCNA. (A) Cluster dendogram tree of module eigengenes. Red line indicates the cutoff height (0.25). TCGA data module dendogram has no outlier modules (left). GEO data module dendogram has the blue and brown modules as outliers (right). (B) Dendogram of DEGs by hierarchical clustering with TOM dissimilarity (upper part). Dendogram of the modules with their assigned colors before and after module merging; TCGA (right) and GEO (left). (C) Heatmap plots of the relationships between merged modules and LGG clinical traits derived from TCGA (right) and GEO (left). In each cell, statistical significance is indicated by the Pearson correlation coefficient and *p*-value. Red and blue color denotes a positive and negative correlation, respectively.

The 4,496 common DEGs of the 529 TCGA samples, as well as the 8,049 common DEGs of the 34 GEO samples, were used for the construction of co-expression networks by WGCNA. To this end, first a soft-thresholding power was chosen by taking into consideration the scale-free network topology criterion; a soft-thresholding power of six for the TCGA samples and four for the GEO samples (Figs. S3 and S4). A dendogram of DEGs was generated by using the TOM-based dissimilarity and hierarchical clustering (Fig. S5). Then, by setting the cutoff height at 0.25, outliers within the detected modules were merged (Fig. 4A). Branches within the dendogram represent modules, and each module is denoted by an assigned color. Genes are represented by short vertical lines within leaves. WGCNA resulted to seven modules based on TCGA data (Fig. 4B, left): blue (223), brown (186), grey (973), red (39), turquoise (1,073), and yellow (140). On the other hand, WGCNA resulted to twenty modules based on GEO data (Fig. 4B, right): black (191), blue (1,096), brown (523), cyan (79), green (337), greenyellow (114), grey (75), grey60 (58), lightcyan (74), lightgreen (49), lightyellow (38), magenta (142), midnightblue (79), pink (162), purple (137), red (270), royalblue (35), salmon (101), tan (102), turquoise (1,147), and yellow (457); the number of genes within each module is shown into parentheses.

### Associations of detected modules with clinical traits of LGG

The investigated clinically significant traits of LGG samples, from both TCGA and GEO, are described below:

- Age at Diagnosis: Age the patient was diagnosed based on the number of years.
- Age: Age of patients.
- Gender: Sex of the patient (male or female).
- IDH Mutant Status: If patients carry a mutation in the *IDH* gene.
- Initial Tumor Pathology: Initial state of the tumor pathology.
- Patient Initial Grade: Glioma grade that the patient was diagnosed.
- Primary Diagnosis: Date of diagnosis based on the number of days.
- Prior Treatment: Information about early treatments after diagnosis.
- Progressed Tumor Pathology: Current stage of tumor pathology.
- Race: Ethnic group of the LGG patients.
- Sample Type: Type of material extracted from regions of the brain.
- Site of Resection Biopsy: Part of the brain that the resection was performed.
- Tissue Organ Origin: Origin of the primary infected organ/tissue.
- Vital Status: Current vital status of the patient (alive or dead).

In our study, those modules significantly correlated with clinical traits based on a Spearman correlation coefficient above 0.3 and *p*-value less than 0.05 were detected. Thus, two modules (blue and yellow) comprised of 363 genes, in TCGA samples (Fig. 4C, right), as well as seven modules (salmon, blue, green, cyan, pink, lightgreen, red) including 2,617 genes, in GEO samples (Fig. 4C, left), are significantly associated with clinicopathological traits. The genes contained in each of those modules were selected for further analysis. There was also a 17% overlap between the clinically related DEGs of TCGA and GEO with respect to the TCGA-derived DEGs.

### LGG molecular networks

**Figure 5:**
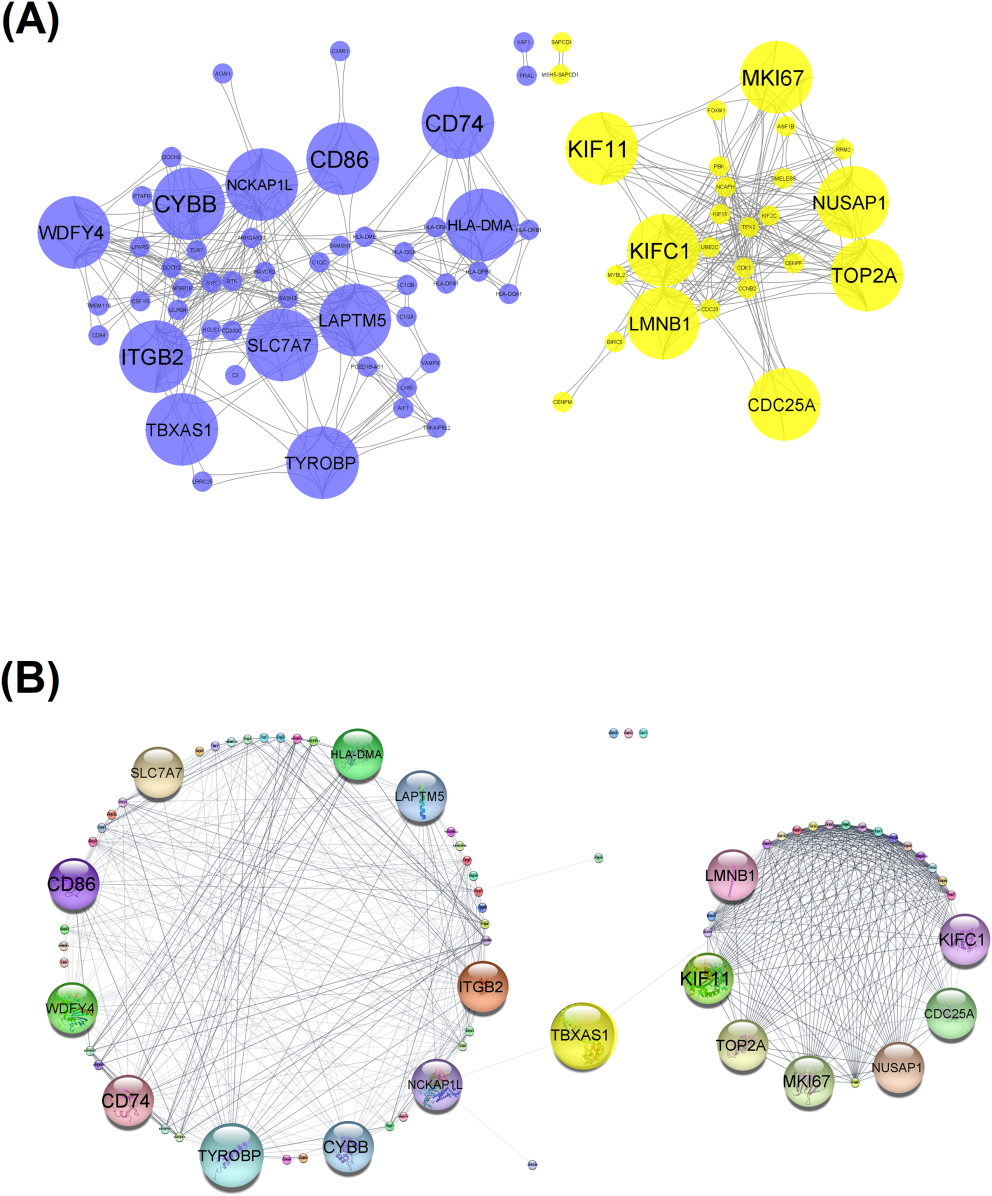
Gene co-expression and protein-protein interaction networks. (A) Co-expression network of TCGA DEGs found in clinically significant modules. The detected hub genes are represented by bigger nodes. Blue and yellow color indicates DEGs from the blue and yellow module, respectively. (B) Network analysis of the associations of the protein products of the clinically significant TCGA DEGs. Proteins are clustered into two distinct clusters and the product of the *TBXAS1* gene acts as an intermodular node.

To detect the most biologically important LGG-relevant genes/gene products, the associations among them were examined by network-based methods For this purpose, the genes comprising the modules associated with clinically significant characteristics were loaded to the Cytoscape platform in order to construct co-expression and protein-protein interaction networks. The gene co-expression network analysis resulted to 76 nodes/genes and 522 edges/interactions (Fig. 5A). There was a total of 35 hub genes with a node degree greater than 10. The STRING protein search option of Cytoscape resulted to a total of 73 nodes/proteins and 558 edges/interactions (Fig. 5B). Based on PPI network analysis, there are 38 hub proteins, the node degree of which was greater than 10. Finally, by combining the results of both networks, eighteen significant common hub genes were detected, namely, *CD74, CD86, CDC25A, CYBB, HLA-DMA, ITGB2, KIF11, KIFC1, LAPTM5, LMNB1, MKI67, NCKAP1L, NUSAP1, SLC7A7, TBXAS1, TOP2A, TYROBP*, and *WDFY4*. All detected hub genes were found to be up-regulated in the TCGA-derived LGG samples.

### Prognostic potential of LGG hub genes

**Figure 6:**
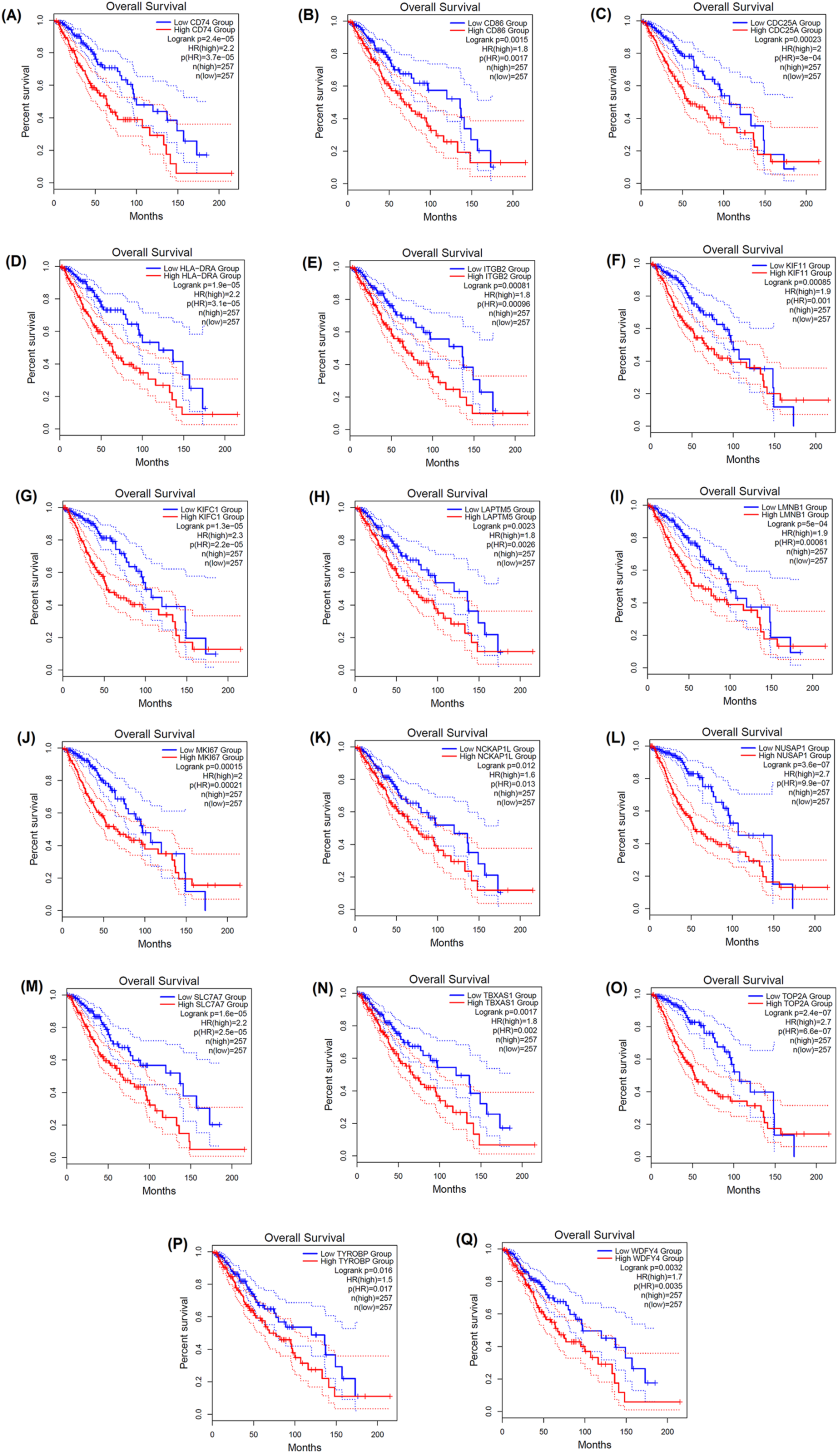
GEPIA-based Kaplan–Meier curves depicting the prognostic significance of seventeen signature genes. (A) *CD74*, (B) *CD86*, (C) *CDC25A*, (D) *HLA-DRA*, (E) *ITGB2*, (F) *KIF11*, (G) *KIFC1*, (H) *LAPTM5*, (I) *LMNB1*, (J) *MKI67*, (K) *NCKAP1L*, (L) *NUSAP1*, (M) *SLC7A7*, (N) *TBXAS1*, (O) *TOP2A*, (P) *TYROBP* and (Q) *WDFY4* for overall survival in LGG patients. The HR “HR(high)” and the corresponding *p*-values “p(HR)” are indicated. The 95% confidence intervals (CI) are denoted by dotted lines. The number of high-risk and low-risk LGG patient groups are represented by “n(high)” and “n(low),” respectively.

**Figure 7:**
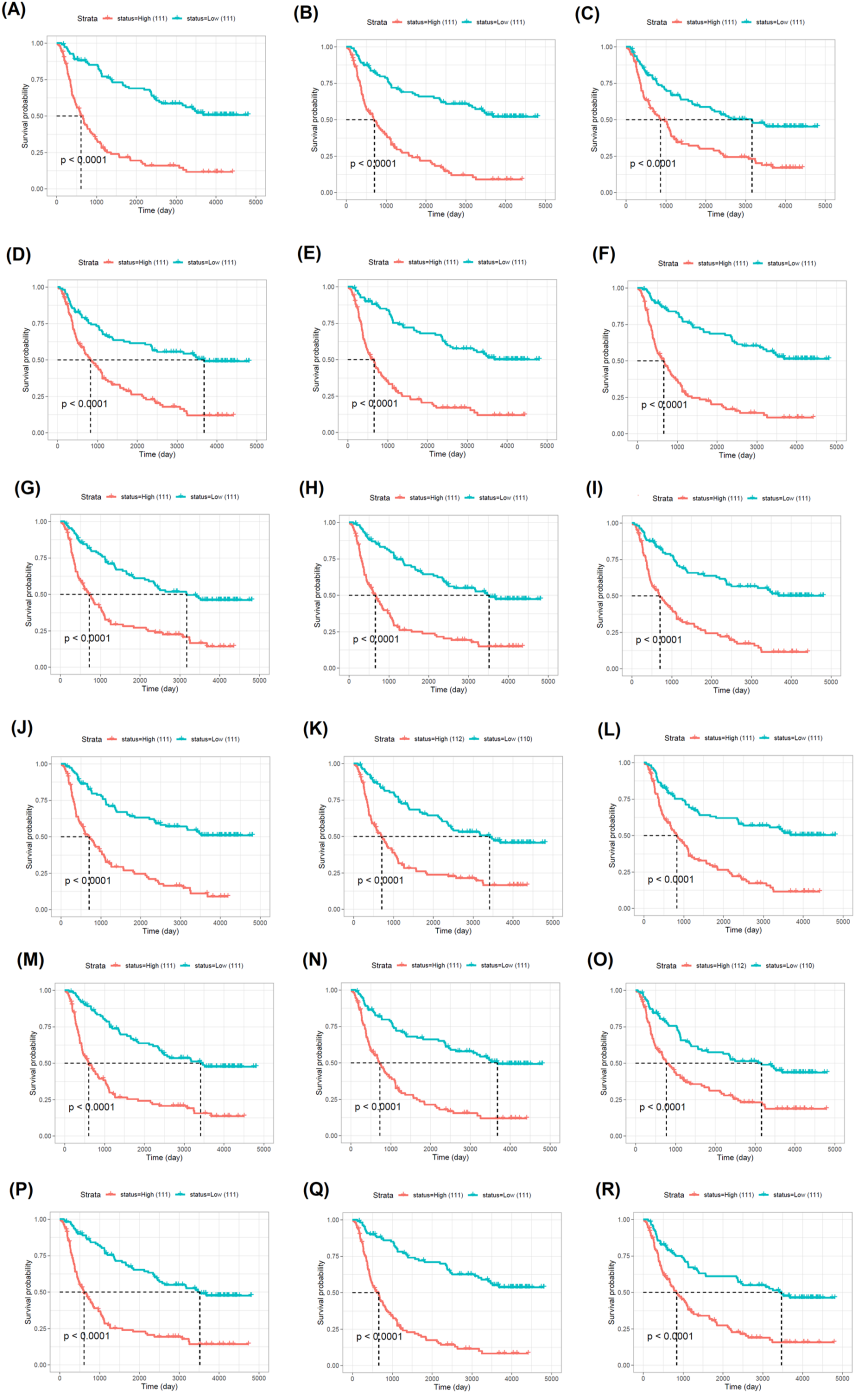
CGGA-based Kaplan–Meier curves illustrating the prognostic value of eighteen signature genes. (A) *CD74*, (B) *CD86*, (C) *CDC25A*, (D) *CYBB*, (E) *HLA-DRA*, (F) *ITGB2*, (G) *KIF11*, (H) *KIFC1*, (I) *LAPTM5*, (J) *LMNB1*, (K) *MKI67*, (L) *NCKAP1L*, (M) *NUSAP1*, (N) *SLC7A7*, (O) *TBXAS1*, (P) *TOP2A*, (Q) *TYROBP* and (R) *WDFY4* for overall survival in primary glioma patients. The number of glioma patients with “High” and “Low” expression of the target gene is shown in parentheses.

Moreover, the impact of these hubs on the overall survival of LGG patients was investigated. The elevated expression of the *CD74, CD86, CDC25A, CYBB, HLA-DRA, ITGB2, KIF11, KIFC1, LAPTM5, LMNB1, MKI67, NCKAP1L, NUSAP1, SLC7A7, TBXAS1, TOP2A, TYROBP* and *WDFY4* hub genes was found to be significantly associated with unfavorable overall survival in LGG patients, as indicated by hazard ratio (HR) values above 1 and *p*-values < 0.05. For *CYBB*, a HR value of 1.3 was not statistically significant (*p* = 0.14) (Fig. 6). Therefore, LGG patients with enhanced expression of these pivotal genes have an increased mortality risk, and may die at a higher rate per unit time, as compared to patients with decreased expression of the corresponding genes. A trend of statistically significant (*p* < 0.05) lower survival probability correlated with higher expression of all eighteen genes was also observed in the survival curves of the Chinese primary glioma patients (Fig. 7).

## Discussion

Gliomas are tumors that originate from glial cells which support the central nervous system (Forst et al., 2014; Wirsching & Weller, 2016). Due to the heterogeneity of gliomas, diagnosis is difficult, even for the experienced medical doctors. Scanned images of brain can be even confusing because some types of gliomas are quite similar. Therefore, glioma treatment options become highly debatable. In the case of misdiagnosis, treatments may result to decreased quality of life, due to after-treatment effects, or even reduced life expectancy (Carabenciov & Buckner, 2019; Tom et al., 2019). The aim of this study was to detect potential diagnostic and prognostic biomarkers between LGG-derived gene expression data and matching normal tissue.

In this way, by exploiting high-throughput transcriptomic data, we identified eighteen ‘hub’ genes with a node degree greater than ten, which represents the maximum number of those connections involving the minimum number of interconnected nodes (*i.e*., genes or proteins). This is because our goal was to select an optimal number of nodes that could represent a panel of genes, which can potentially discriminate LGG from normal brain tissue. Of those, *CD74* codes for a protein that regulates antigen presentation to immune cells. In general, *CD74* was demonstrated to play a role in the development of many types of cancers. Xu et al. (2021) revealed that *CD74* expression was higher in glioma cells compared to normal brain cells. Moreover, the expression of *CD74* was higher in HGG compared to LGG. It was suggested that *CD74* is a biomarker of LGG for poor prognosis and also could be a therapeutic target for glioma (Xu et al., 2021). In another study, expression of *CD74* was shown to contribute to resistance to treatments (*e.g*., themozolomide) in GBM (Presti et al., 2018). In our study, *CD74* was found to be up-regulated in the TCGA-LGG samples.

*CD86* is a protein-coding gene, expressed by antigen presenting cells. In a recent study, Ahmed et al. (2022) suggested that *CD86* may actually serve as a potential biomarker for the prognosis of GBM and found that *CD86* expression swas high in GBM patients. Also, Qiu et al. (2021) found that *CD86* overexpression is an unfavorable marker for LGG prognosis, as in our study.

*CYBB*, found to be overexpressed both in TCGA- and GEO-LGG samples, encodes the beta chain of cytochrome which has a crucial role in ion channels. In a study, where screening for biomarkers of LGG was performed, *CYBB* was shown to be one of the key DEGs (Guo et al., 2022).

Although *HLA-DMA* was previously associated with breast cancer, it is also known to have a role in cancer progression and resistance to drugs. Yin et al. (2022) suggested *HLA-DMA* to have a prognostic value in GBM. We also found *HLA-DMA* to be up-regulated in the TCGA-LGG samples and represent a potential prognostic marker of LGG.

*ITGB2* codes for the beta chain of an integrin heterodimer. Integrins are basically involved in cell surface-related functions such as cell adhesion or signaling. In a recent study, Xu et al. (2022) assessed the prognostic potential of *ITGB2* in glioma patients. They found that the glioma grade increases relatively to the expression level of *ITGB2*, and suggested that *ITGB2* could represent a novel predictor for glioma (Xu et al., 2022). Li et al. (2021b), in an effort to detect hub genes in LGG by using data derived from the TCGA and CGGA, found *ITGB2* to be a key gene. Including *ITGB2*, several key genes of this study are also similar to the ones identified in our work (Li et al., 2021b).

*KIF11*, shown to be up-regulated in LGG, encodes a motor protein which plays a role in the spindle dynamics of cells. A study about the inhibitory effects of *KIF11* on mice revealed that *KIF11* is up-regulated in GBM, specifically in proliferating and migrating cells (Venere et al., 2015). One important reason that *KIF11* represents a suitable target in GBM is that its mostly explored inhibitors are non neurotoxic (Gampa et al., 2020). Liu et al. (2022) found that *KIF11* was up-regulated in gliomas and, also, was negatively associated with the survival of patients, based on TCGA-derived data. Moreover, it was suggested that *KIF11* is necessary for the stemness of glioma cells and thereby cell proliferation.

*KIFC1*, which encodes a kinesin motor known to have a role in the clustering of the centrosomes of cancer cells, is involved in several types of cancers. Wu et al. (2021) found that there is elevated expression of *KIFC1* in GBM and its inhibition suppressed proliferation and resistance to temozolomide. Dai et al. (2017) also showed that *KIFC1* is up-regulated in grade III gliomas, like in our study.

*LAPTM5* encodes protein E3, which is a transmembrane receptor. There are controversial findings regarding the role of *LAPTM5* in gliomas. In one study, *LAPTM5* was suggested to be a biomarker of poor prognosis in GBMs (Hajj et al., 2020). On the other hand, in another study, *LAPTM5* was found to act as a tumor suppressor and its inhibition actually promoted invasiveness of GBMs based on *in vitro* and *in vivo* experiments (Berberich et al., 2020). Nevertheless, in our study we found *LAPTM5* to be up-regulated in LGG and also to be a poor prognostic marker for the overall survival of LGG patients.

The product of *LMNB1* is one of the three component proteins of nuclear lamina. In a recent study based on microarray data, it was shown that *LMNB1* expression was higher in gliomas, and was also related with poor survival rates. Moreover, inhibition of *LMNB1* suppressed glioma proliferation (Zhou et al., 2021). Pei et al. (2022) showed that *LMNB1* is up-regulated in glioma cells, and overexpression of *lamin* genes causes abnormalities in human astrocytes which are actually glial cells that support the central nervous system.

*NCKAP1L*, found to be up-regulated in our study, was also shown to constitute a hub gene in LGG based on TCGA- and CGGA-derived data. In the same study, the genes *LAMC1*, *CD74*, *HLA-DMA*, *ITGB2*, *TYROBP*, *LAPTM5*, and *CYBB* (Su et al., 2019), which were also identified in our work, were shown to constitute hub genes.

*SLC7A7* belongs to the family of *solute carrier* seven genes, which play a role in nutrition intake as transporters. In a study, where the genes of this family were examined in LGG by using TCGA-derived data, it was shown that *SLCA7A* was significantly up-regulated in patients with LGG and was also a poor prognostic marker (Liu et al., 2021a), similarly to the findings of our study. Moreover, Fan et al. (2013) examined a Chinese population of 736 glioma patients and 793 normal subjects and found that polymorphisms in *SLC7A7* actually contribute to the heterogeneity of gliomas.

*TBXAS1* codes for a member of the cytochrome P450 superfamily. These proteins are implicated in drug metabolism and also the synthesis of fat molecules. Elevated expression of *TBXAS1* was found in diffuse LGG patients, which was also associated with poor overall survival (Wang et al., 2017), like in our study.

The product of *TOP2A* is a DNA topoisomerase with a critical role in transcription. A novel variant of *TOP2A* was identified in GBM patients, which was associated with altered transcriptional regulation and decreased survival (Gielniewski et al., 2020). Zhou et al. (2018) revealed a relationship between *TOP2A* up-regulation and poor prognosis in gliomas, and also suggested that *MKI67*, another hub gene found to be up-regulated in our study, is correlated with *TOP2A* overexpression.

*TYROBP* encodes a transmembrane signaling protein. In a study by Lu et al. (2021), where glioma datasets derived from Oncomine, GEPIA2 and CGGA were examined, *TYROBP* was found to be one of the hub genes up-regulated in LGG. Their results also revealed that TYROBP was implicated in pivotal signaling pathways, such as JAK-STAT, suggesting that TYROBP could actually contribute largely to LGG through its involvement in signaling networks (Lu et al., 2021).

*WDFY4*, found to be up-regulated both in TCGA- and GEO-LGG samples, is implicated in autophagy and also the processing and cross-presentation of viral and cancer antigens by dendritic cells (Theisen et al., 2018).

Symptoms of brain tumors, with headache being the most common one, are generally considered to be either non-significant or possibly benign tumors, thereby leading to poor diagnosis and prognosis. It has been demonstrated that blood tests can be used for the timely and accurate detection of circulating tumor cells (Macarthur et al., 2014; Jelski & Mroczko, 2021). In particular, Butler et al. (2019) tested a methodology, called ATR-FTIR, on blood tests, and successfully differentiated brain cancer and normal cells with a 93.2% sensitivity and 92.8% specificity. In another study, Podnar et al. (2019) used machine learning on the blood tests of patients with brain tumors in order to investigate any differences with 96% sensitivity and 74% specificity. In a similar manner, the eighteen signature genes identified in this study could be tested for their diagnostic potential in patients with brain neoplasms through blood tests.

## Conclusions

In this study, an integrative *in silico* approach was applied, including differential gene co-expression analysis and network-based methods, in order to identify gene expression signatures in LGG. Eighteen hub genes were detected, all up-regulated in LGG, and significantly associated with unfavorable prognosis in LGG patients. Their importance in LGG was also assessed on the basis of their relationship with LGG clinical traits. Hence, these genes could be taken into consideration in the clinical setting as candidate diagnostic or prognostic biomarkers for the accurate, timely and cost-effective diagnosis of LGG, and for monitoring LGG patients’ progression. The findings of this study could lay the foundation for further *in vitro* and *in vivo* experimental studies towards the elucidation of the underlying mechanisms of low-grade glioma genesis.

## Supplemental Information

10.7717/peerj.15096/supp-1 Supplemental Information 1 List of genes differentially expressed between the TCGA-LGG and GEO-LGG and the corresponding normal GTEx-derived samples.

10.7717/peerj.15096/supp-2 Supplemental Information 2 Genes differentially expressed between TCGA-LGG and normal samples. Volcano plots of detected DEGs generated by edgeR (left), DESeq2 (middle) and limma (right). Vertical dash lines indicate log2(FC) and horizontal dash line indicates –log10(0.05). Red and blue dots correspond to up-regulated and down-regulated genes, respectively, and gray dots represent insignificant genes.

10.7717/peerj.15096/supp-3 Supplemental Information 3 Genes differentially expressed between GEO-LGG and normal samples. Volcano plots of detected DEGs generated by edgeR (left), DESeq2 (middle) and limma (right). Vertical dash lines indicate log2(FC) and horizontal dash line indicates –log10(0.05). Red and blue dots correspond to up-regulated and down-regulated genes, respectively, and gray dots represent insignificant genes.

10.7717/peerj.15096/supp-4 Supplemental Information 4 Scale independence plot and Mean Connectivity plot of TCGA common DEGs used for selecting soft-thresholding power.

10.7717/peerj.15096/supp-5 Supplemental Information 5 Scale independence plot and Mean Connectivity plot of GEO common DEGs used for selecting soft-thresholding power.

10.7717/peerj.15096/supp-6 Supplemental Information 6 Heatmap of the TOM matrix of TCGA (left) and GEO (right). The outer layer is the dendogram tree of each gene. The middle part is the merged modules with their assigned colors. In the inner heatmap, lighter and darker color indicates higher and lower topological overlaps, respectively.

## Additional Information and Declarations

### Data Availability

The following information was supplied regarding data availability: The study uses publicly available sequencing datasets.

## References

[ref-1] Ahmed MH, Hernandez-Verdin I, Bielle F, Verreault M, Lerond J, Alentorn A, Sanson M, Idbaih A (2022). Expression and prognostic value of CD80 and CD86 in the tumor microenvironment of newly diagnosed glioblastoma. Canadian Journal of Neurological Sciences.

[ref-2] Berberich A, Bartels F, Tang Z, Knoll M, Pusch S, Hucke N, Kessler T, Dong Z, Wiestler B, Winkler F, Platten M, Wick W, Abdollahi A, Lemke D (2020). LAPTM5-CD40 crosstalk in glioblastoma invasion and temozolomide resistance. Frontiers in Oncology.

[ref-3] Butler HJ, Brennan PM, Cameron JM, Finlayson D, Hegarty MG, Jenkinson MD, Palmer DS, Smith BR, Baker MJ (2019). Development of high-throughput ATR-FTIR technology for rapid triage of brain cancer. Nature Communications.

[ref-4] Carabenciov ID, Buckner JC (2019). Controversies in the therapy of low-grade gliomas. Current Treatment Options in Oncology.

[ref-5] Chen R, Cohen AL, Colman H (2016). Targeted therapeutics in patients with high-grade gliomas: past, present, and future. Current Treatment Options in Oncology.

[ref-6] Chin CH, Chen SH, Wu HH, Ho CW, Ko MT, Lin CY (2014). cytoHubba: identifying hub objects and sub-networks from complex interactome. BMC Systems Biology.

[ref-7] Claus EB, Walsh KM, Wiencke JK, Molinaro AM, Wiemels JL, Schildkraut JM, Bondy ML, Berger M, Jenkins R, Wrensch M (2015). Survival and low-grade glioma: the emergence of genetic information. Neurosurgical Focus.

[ref-8] Colaprico A, Silva TC, Olsen C, Garofano L, Cava C, Garolini D, Sabedot TS, Malta TM, Pagnotta SM, Castiglioni I, Ceccarelli M, Bontempi G, Noushmehr H (2016). TCGAbiolinks: an R/Bioconductor package for integrative analysis of TCGA data. Nucleic Acids Research.

[ref-9] Collado-Torres L, Nellore A, Kammers K, Ellis SE, Taub MA, Hansen KD, Jaffe AE, Langmead B, Leek JT (2017). Reproducible RNA-seq analysis using recount2. Nature Biotechnology.

[ref-10] Dai J, Bing Z, Zhang Y, Li Q, Niu L, Liang W, Yuan G, Duan L, Yin H, Pan Y (2017). Integrated mRNAseq and microRNAseq data analysis for grade III gliomas. Molecular Medicine Reports.

[ref-11] Delgado-Lopez PD, Corrales-Garcia EM, Martino J, Lastra-Aras E, Duenas-Polo MT (2017). Diffuse low-grade glioma: a review on the new molecular classification, natural history and current management strategies. Clinical & Translational Oncology.

[ref-12] Dobin A, Davis CA, Schlesinger F, Drenkow J, Zaleski C, Jha S, Batut P, Chaisson M, Gingeras TR (2013). STAR: ultrafast universal RNA-seq aligner. Bioinformatics.

[ref-13] Dono A, Ballester LY, Primdahl D, Esquenazi Y, Bhatia A (2021). IDH-mutant low-grade glioma: advances in molecular diagnosis, management, and future directions. Current Oncology Reports.

[ref-14] Edgar R, Domrachev M, Lash AE (2002). Gene expression omnibus: NCBI gene expression and hybridization array data repository. Nucleic Acids Research.

[ref-15] Fan S, Zhao Y, Li X, Du Y, Wang J, Song X, Zhou F, Chen H, Chen G, Zhao Y, Mao Y, Lan Q (2013). Genetic variants in SLC7A7 are associated with risk of glioma in a Chinese population. Experimental Biology and Medicine.

[ref-16] Ferris SP, Hofmann JW, Solomon DA, Perry A (2017). Characterization of gliomas: from morphology to molecules. Virchows Archiv.

[ref-17] Forst DA, Nahed BV, Loeffler JS, Batchelor TT (2014). Low-grade gliomas. The Oncologist.

[ref-18] Frankish A, Diekhans M, Jungreis I, Lagarde J, Loveland J E, Mudge JM, Sisu C, Wright JC, Armstrong J, Barnes I, Berry A, Bignell A, Boix C, Carbonell Sala S, Cunningham F, Di Domenico Tás, Donaldson S, Fiddes I T, García Girón C, Gonzalez JM, Grego T, Hardy M, Hourlier T, Howe KL, Hunt T, Izuogu OG, Johnson R, Martin FJ, Martínez L, Mohanan S, Muir P, Navarro FCP, Parker A, Pei B, Pozo F, Riera FC, Ruffier M, Schmitt BM, Stapleton E, Suner M-M, Sycheva I, Uszczynska-Ratajczak B, Wolf MY, Xu J, Yang Y T, Yates A, Zerbino D, Zhang Y, Choudhary J S, Gerstein M, Guigó R, Hubbard TJP, Kellis M, Paten B, Tress ML, Flicek P (2021). Gencode 2021. Nucleic Acids Research.

[ref-19] Gampa G, Kenchappa RS, Mohammad AS, Parrish KE, Kim M, Crish JF, Luu A, West R, Hinojosa AQ, Sarkaria JN, Rosenfeld SS, Elmquist WF (2020). Enhancing brain retention of a KIF11 inhibitor significantly improves its efficacy in a mouse model of glioblastoma. Scientific Reports.

[ref-20] Gielniewski B, Poleszak K, Roura A-J, Szadkowska P, Krol SK, Guzik R, Wiechecka P, Maleszewska M, Kaza B, Marchel A, Czernicki T, Koziarski A, Zielinski G, Styk A, Kawecki M, Szczylik C, Czepko R, Banach M, Kaspera W, Szopa W, Bujko M, Czapski B, Zabek M, Izycka-Swieszewska E, Kloc W, Nauman P, Cieslewicz J, Wojtas B, Kaminska B (2020). The novel, recurrent mutation in the TOP2A gene results in the enhanced topoisomerase activity and transcription deregulation in glioblastoma. BioRxiv.

[ref-21] GTEx Consortium (2013). The genotype-tissue expression (GTEx) project. Nature Genetics.

[ref-22] Guo F, Yan J, Ling G, Chen H, Huang Q, Mu J, Mo L (2022). Screening and identification of key biomarkers in lower grade glioma via bioinformatical analysis. Applied Bionics and Biomechanics.

[ref-23] Hajj GNM, Silva FF, de Bellis B, Lupinacci FCS, Bellato HM, Cruz JR, Segundo CNC, Faquini IV, Torres LC, Sanematsu PI, Begnami MD, Martins VR, Roffé M (2020). Aberrant expression of RSK1 characterizes high-grade gliomas with immune infiltration. Molecular Oncology.

[ref-24] Jelski W, Mroczko B (2021). Molecular and circulating biomarkers of brain tumors. International Journal of Molecular Sciences.

[ref-25] Jolliffe IT, Cadima J (2016). Principal component analysis: a review and recent developments. Philosophical Transactions Series A, Mathematical, Physical, and Engineering Sciences.

[ref-70] Kassambara A, Mundt F (2020). factoextra: extract and visualize the results of multivariate data Analyses. https://CRAN.R-project.org/package=factoextra.

[ref-67] Kolde R (2019). https://cran.r-project.org/web/packages/pheatmap/index.html.

[ref-26] Kumthekar P, Raizer J, Singh S (2015). Low-grade glioma. Cancer Treatment and Research.

[ref-27] Langfelder P, Horvath S (2008). WGCNA: an R package for weighted correlation network analysis. BMC Bioinformatics.

[ref-28] Leinonen R, Sugawara H, Shumway M, International Nucleotide Sequence Database Collaboration (2011). The sequence read archive. Nucleic Acids Research.

[ref-29] Li B, Dewey CN (2011). RSEM: accurate transcript quantification from RNA-seq data with or without a reference genome. BMC Bioinformatics.

[ref-30] Li C, Tang Z, Zhang W, Ye Z, Liu F (2021a). GEPIA2021: integrating multiple deconvolution-based analysis into GEPIA. Nucleic Acids Research.

[ref-31] Li G, Wu Z, Gu J, Zhu Y, Zhang T, Wang F, Huang K, Gu C, Xu K, Zhan R, Shen J (2021b). Metabolic signature-based subtypes may pave novel ways for low-grade glioma prognosis and therapy. Frontiers in Cell and Developmental Biology.

[ref-32] Liu W, Ji Y, Ren R, Zhang G (2021a). The solute carrier family 7 genes are potential diagnostic and prognostic biomarkers in lower grade glioma. Research Square preprint.

[ref-33] Liu J, Lichtenberg T, Hoadley KA, Poisson LM, Lazar AJ, Cherniack AD, Kovatich AJ, Benz CC, Levine DA, Lee AV, Omberg L, Wolf DM, Shriver CD, Thorsson V, Hu H, Cancer Genome Atlas Research Network (2018). An integrated TCGA pan-cancer clinical data resource to drive high-quality survival outcome analytics. Cell.

[ref-34] Liu B, Zhang G, Cui S, Du G (2022). Upregulation of KIF11 in TP53 mutant glioma promotes tumor stemness and drug resistance. Cellular and Molecular Neurobiology.

[ref-35] Liu W, Zou J, Ren R, Liu J, Zhang G, Wang M (2021b). A novel 10-gene signature predicts poor prognosis in low grade glioma. Technology in Cancer Research & Treatment.

[ref-36] Love MI, Huber W, Anders S (2014). Moderated estimation of fold change and dispersion for RNA-seq data with DESeq2. Genome Biology.

[ref-37] Lu J, Peng Y, Huang R, Feng Z, Fan Y, Wang H, Zeng Z, Ji Y, Wang Y, Wang Z (2021). Elevated TYROBP expression predicts poor prognosis and high tumor immune infiltration in patients with low-grade glioma. BMC Cancer.

[ref-38] Macarthur KM, Kao GD, Chandrasekaran S, Alonso-Basanta M, Chapman C, Lustig RA, Wileyto EP, Hahn SM, Dorsey JF (2014). Detection of brain tumor cells in the peripheral blood by a telomerase promoter-based assay. Cancer Research.

[ref-39] Pei S, Wang X, Wang X, Huang H, Tao H, Xie B, Yang A, Qiu M, Tan Z (2022). Aberrant nuclear lamina contributes to the malignancy of human gliomas. Journal of Genetics and Genomics.

[ref-40] Podnar S, Kukar M, Guncar G, Notar M, Gosnjak N, Notar M (2019). Diagnosing brain tumours by routine blood tests using machine learning. Scientific Reports.

[ref-41] Presti M, Mazzon E, Basile MS, Petralia MC, Bramanti A, Colletti G, Bramanti P, Nicoletti F, Fagone P (2018). Overexpression of macrophage migration inhibitory factor and functionally-related genes, D-DT, CD74, CD44, CXCR2 and CXCR4, in glioblastoma. Oncology Letters.

[ref-42] Qiu H, Tian W, He Y, Li J, He C, Li Y, Liu N, Li J (2021). Integrated analysis reveals prognostic value and immune correlates of CD86 expression in lower grade glioma. Frontiers in Oncology.

[ref-68] R Core Team (2022). R: A language and environment for statistical computing. Version 4.2.0. https://www.r-project.org.

[ref-43] Ren X, Kuan PF (2020). RNAAgeCalc: a multi-tissue transcriptional age calculator. PLOS ONE.

[ref-44] Reon BJ, Anaya J, Zhang Y, Mandell J, Purow B, Abounader R, Dutta A (2016). Expression of lncRNAs in low-grade gliomas and glioblastoma multiforme: an in silico analysis. PLOS Medicine.

[ref-45] Risso D, Schwartz K, Sherlock G, Dudoit S (2011). GC-content normalization for RNA-seq data. BMC Bioinformatics.

[ref-46] Ritchie ME, Phipson B, Wu D, Hu Y, Law CW, Shi W, Smyth GK (2015). limma powers differential expression analyses for RNA-sequencing and microarray studies. Nucleic Acids Research.

[ref-47] Robinson MD, McCarthy DJ, Smyth GK (2010). edgeR: a bioconductor package for differential expression analysis of digital gene expression data. Bioinformatics.

[ref-48] Rossi M, Sani S, Nibali MC, Fornia L, Bello L, Byrne RW (2019). Mapping in low-grade glioma surgery: low- and high-frequency stimulation. Neurosurgery Clinics of North America.

[ref-49] Schiff D (2017). Low-grade gliomas. Continuum.

[ref-50] Shannon P, Markiel A, Ozier O, Baliga NS, Wang JT, Ramage D, Amin N, Schwikowski B, Ideker T (2003). Cytoscape: a software environment for integrated models of biomolecular interaction networks. Genome Research.

[ref-51] Su J, Long W, Ma Q, Xiao K, Li Y, Xiao Q, Peng G, Yuan J, Liu Q (2019). Identification of a tumor microenvironment-related eight-gene signature for predicting prognosis in lower-grade gliomas. Frontiers in Genetics.

[ref-52] Szklarczyk D, Gable AL, Nastou KC, Lyon D, Kirsch R, Pyysalo S, Doncheva NT, Legeay M, Fang T, Bork P, Jensen LJ, von Mering C (2021). The STRING database in 2021: customizable protein-protein networks, and functional characterization of user-uploaded gene/measurement sets. Nucleic Acids Research.

[ref-53] Tang Z, Li C, Kang B, Gao G, Li C, Zhang Z (2017). GEPIA: a web server for cancer and normal gene expression profiling and interactive analyses. Nucleic Acids Research.

[ref-54] Theisen DJ, Davidson JT, Briseño CG, Gargaro M, Lauron EJ, Wang Q, Desai P, Durai V, Bagadia P, Brickner JR, Beatty WL, Virgin HW, Gillanders WE, Mosammaparast N, Diamond MS, Sibley LD, Yokoyama W, Schreiber RD, Murphy TL, Murphy KM (2018). WDFY4 is required for cross-presentation in response to viral and tumor antigens. Science.

[ref-55] Tom MC, Cahill DP, Buckner JC, Dietrich J, Parsons MW, Yu JS (2019). Management for different glioma subtypes: are all low-grade gliomas created equal?. American Society of Clinical Oncology Educational Book.

[ref-56] Venere M, Horbinski C, Crish JF, Jin X, Vasanji A, Major J, Burrows AC, Chang C, Prokop J, Wu Q, Sims PA, Canoll P, Summers MK, Rosenfeld SS, Rich JN (2015). The mitotic kinesin KIF11 is a driver of invasion, proliferation, and self-renewal in glioblastoma. Science Translational Medicine.

[ref-57] Wang S, Jin F, Fan W, Liu F, Zou Y, Hu X, Xu H, Han P (2017). Gene expression meta-analysis in diffuse low-grade glioma and the corresponding histological subtypes. Scientific Reports.

[ref-58] Wirsching HG, Weller M (2016). The role of molecular diagnostics in the management of patients with gliomas. Current Treatment Options in Oncology.

[ref-59] Wu J, Wang X, Yuan X, Shan Q, Wang Z, Wu Y, Xie J (2021). Kinesin family member C1 increases temozolomide resistance of glioblastoma through promoting DNA damage repair. Cell Transplantation.

[ref-60] Xu S, Li X, Tang L, Liu Z, Yang K, Cheng Q (2021). CD74 correlated with malignancies and immune microenvironment in gliomas. Frontiers in Molecular Biosciences.

[ref-61] Xu H, Zhang A, Han X, Li Y, Zhang Z, Song L, Wang W, Lou M (2022). ITGB2 as a prognostic indicator and a predictive marker for immunotherapy in gliomas. Cancer Immunology, Immunotherapy.

[ref-62] Yin X, Wu Q, Hao Z, Chen L (2022). Identification of novel prognostic targets in glioblastoma using bioinformatics analysis. BioMedical Engineering OnLine.

[ref-63] Zhang Y, Parmigiani G, Johnson WE (2020). ComBat-seq: batch effect adjustment for RNA-seq count data. NAR Genomics and Bioinformatics.

[ref-64] Zhao Z, Zhang K-N, Wang Q, Li G, Zeng F, Zhang Y, Wu F, Chai R, Wang Z, Zhang C, Zhang W, Bao Z, Jiang T (2021). Chinese glioma genome atlas (CGGA): a comprehensive resource with functional genomic data from Chinese glioma patients. Genomics, Proteomics & Bioinformatics.

[ref-65] Zhou T, Wang Y, Qian D, Liang Q, Wang B (2018). Over-expression of TOP2A as a prognostic biomarker in patients with glioma. International Journal of Clinical and Experimental Pathology.

[ref-66] Zhou D, Wang M, Zhang Y, Wang K, Zhao M, Wang Y, Wang X, Yu R, Zhou X (2021). Screening and identification of LMNB1 and DLGAP5, two key biomarkers in gliomas. Bioscience Reports.

