# Supplementary figures and images for: An in silico approach to the identification of diagnostic and prognostic markers in low-grade gliomas
Source: PeerJ. 2023 Mar 16;11:e15096. doi: 10.7717/peerj.15096 (PMC10024901; doi:10.7717/peerj.15096)

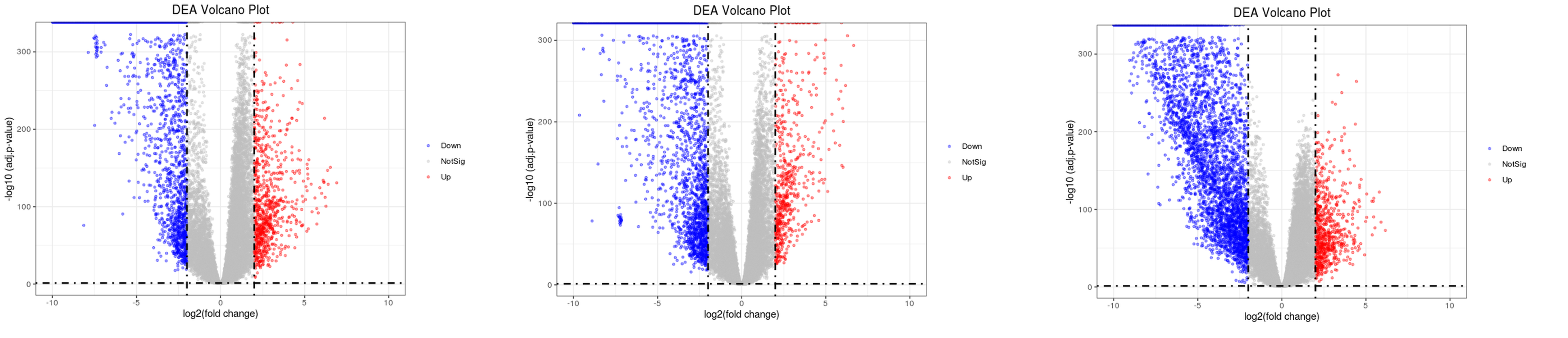

Supplement: Supplemental Information 2 — Volcano plots of detected DEGs generated by edgeR (left), DESeq2 (middle) and limma (right). Vertical dash lines indicate log2(FC) and horizontal dash line indicates –log10(0.05). Red and blue dots correspond to up-regulated and down-regulated genes, respectively, and gray dots represent insignificant genes. [file peerj-11-15096-s002.png]

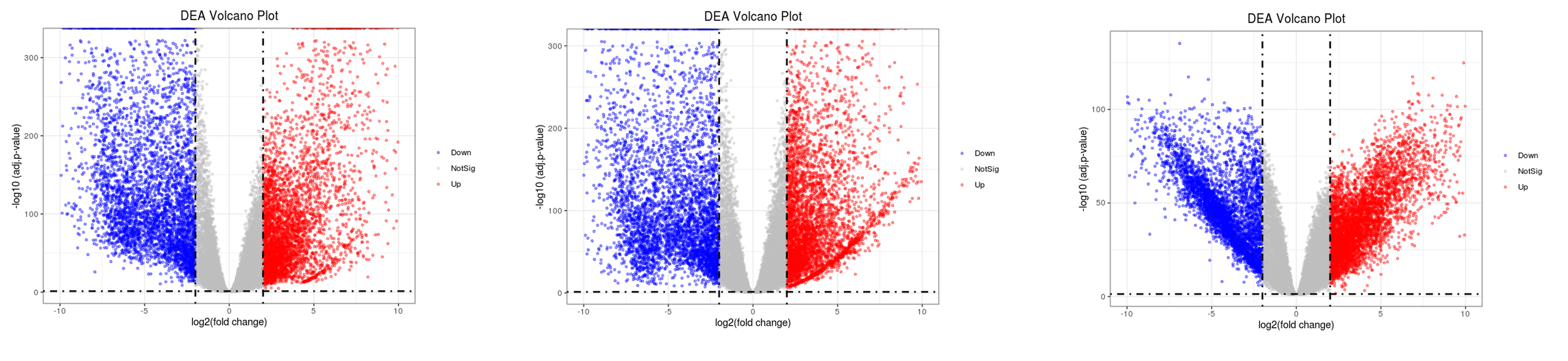

Supplement: Supplemental Information 3 — Vertical dash lines indicate log2(FC) and horizontal dash line indicates –log10(0.05). Red and blue dots correspond to up-regulated and down-regulated genes, respectively, and gray dots represent insignificant genes. [file peerj-11-15096-s003.png]

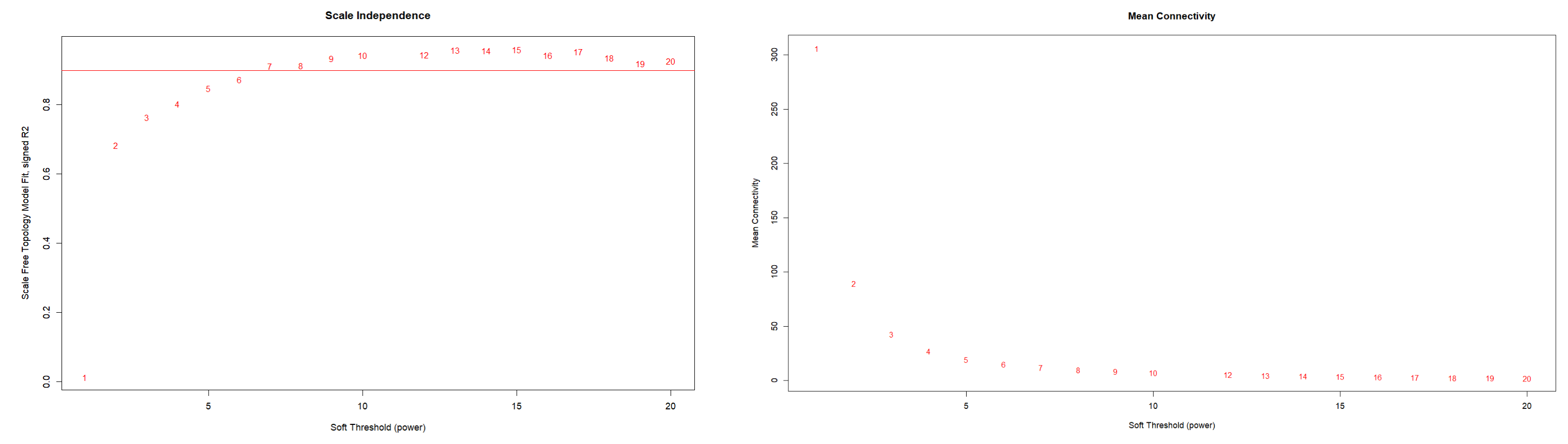

Supplement: Supplemental Information 4 [file peerj-11-15096-s004.png]

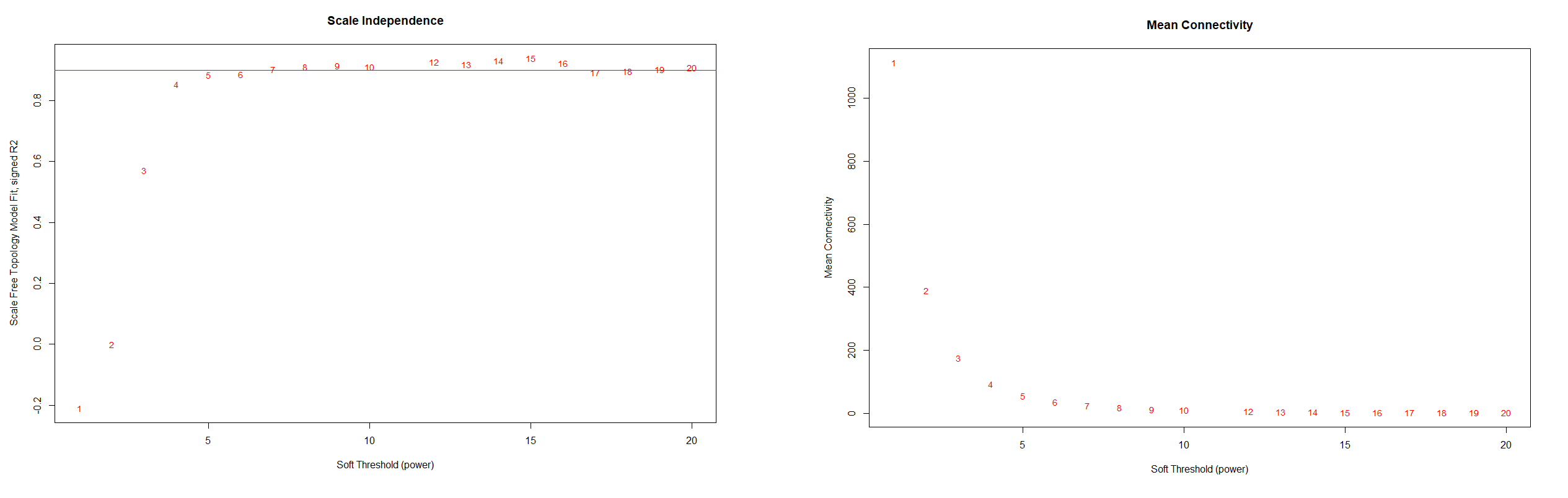

Supplement: Supplemental Information 5 [file peerj-11-15096-s005.png]

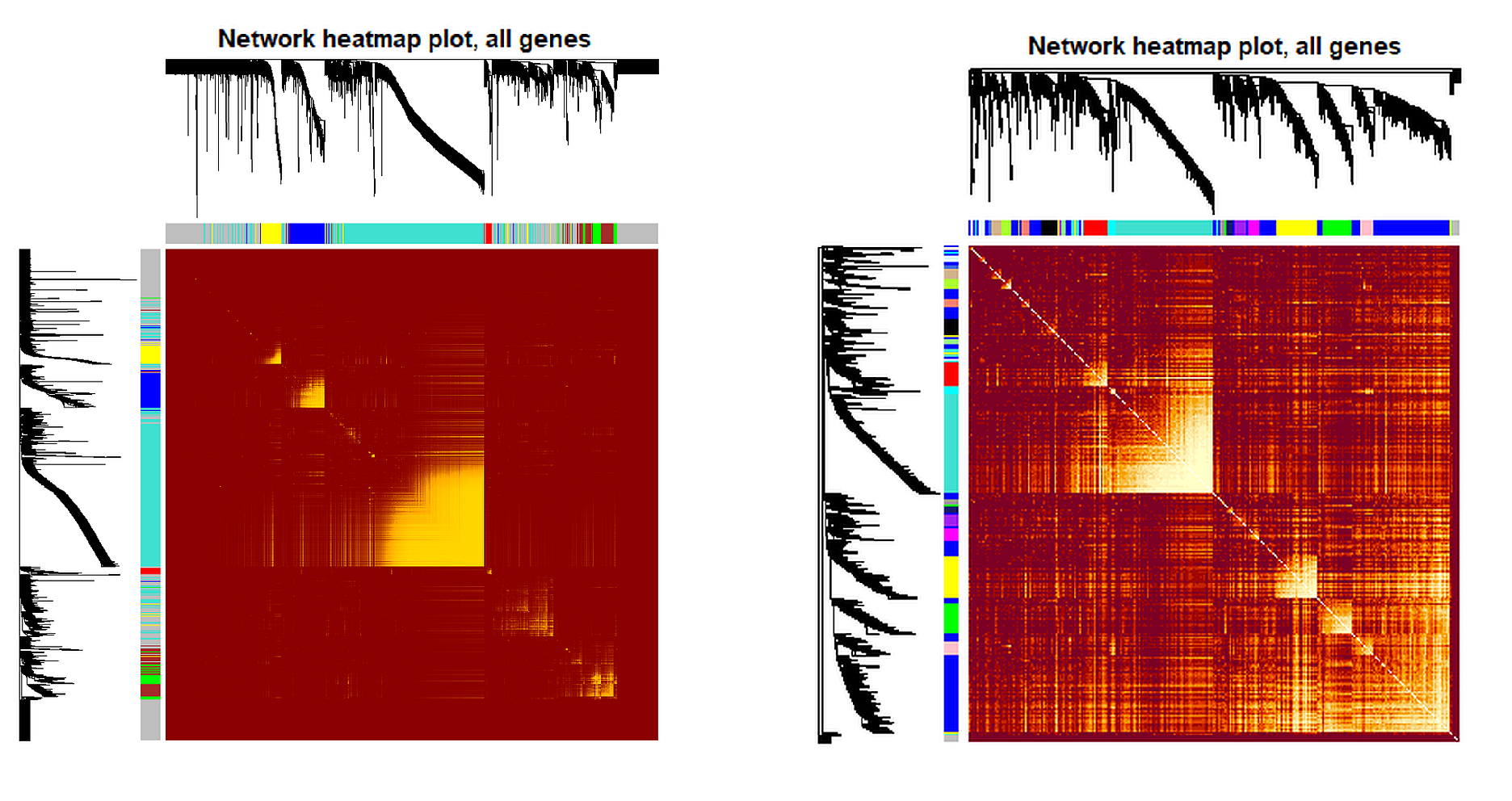

Supplement: Supplemental Information 6 — The outer layer is the dendogram tree of each gene. The middle part is the merged modules with their assigned colors. In the inner heatmap, lighter and darker color indicates higher and lower topological overlaps, respectively. [file peerj-11-15096-s006.png]
